# The Interplay among Acorn Abundance and Rodent Behavior Drives the Spatial Pattern of Seedling Recruitment in Mature Mediterranean Oak Forests

**DOI:** 10.1371/journal.pone.0129844

**Published:** 2015-06-12

**Authors:** Pau Sunyer, Ester Boixadera, Alberto Muñoz, Raúl Bonal, Josep Maria Espelta

**Affiliations:** 1 CREAF, Cerdanyola del Vallès, Catalonia, Spain; 2 Servei d’Estadística Aplicada, Universitat Autònoma de Barcelona, Cerdanyola del Vallès, Catalonia, Spain; 3 Departamento de Didáctica de las Ciencias Experimentales, Facultad de Educación, Universidad Complutense de Madrid, Madrid, Spain; 4 Forest Research Group (GIF) INDEHESA, University of Extremadura, Plasencia, Spain; 5 DITEG Research Group, University of Castilla-La Mancha, Toledo, Spain; University of Missouri Kansas City, UNITED STATES

## Abstract

The patterns of seedling recruitment in animal-dispersed plants result from the interactions among environmental and behavioral variables. However, we know little on the contribution and combined effect of both kinds of variables. We designed a field study to assess the interplay between environment (vegetation structure, seed abundance, rodent abundance) and behavior (seed dispersal and predation by rodents, and rooting by wild boars), and their contribution to the spatial patterns of seedling recruitment in a Mediterranean mixed-oak forest. In a spatially explicit design, we monitored intensively all environmental and behavioral variables in fixed points at a small spatial scale from autumn to spring, as well as seedling emergence and survival. Our results revealed that the spatial patterns of seedling emergence were strongly related to acorn availability on the ground, but not by a facilitationeffect of vegetation cover. Rodents changed seed shadows generated by mother trees by dispersing most seeds from shrubby to open areas, but the spatial patterns of acorn dispersal/predation had no direct effect on recruitment. By contrast, rodents had a strong impact on recruitment as pilferers of cached seeds. Rooting by wild boars also reduced recruitment by reducing seed abundance, but also by changing rodent’s behavior towards higher consumption of acorns in situ. Hence, seed abundance and the foraging behavior of scatter-hoarding rodents and wild boars are driving the spatial patterns of seedling recruitment in this mature oak forest, rather than vegetation features. The contribution of vegetation to seedling recruitment (e.g. facilitation by shrubs) may be context dependent, having a little role in closed forests, or being overridden by directed seed dispersal from shrubby to open areas. We warn about the need of using broad approaches that consider the combined action of environment and behavior to improve our knowledge on the dynamics of natural regeneration in forests.

## Introduction

Seedling recruitment in forests results from the sequential interaction among different processes, from seed production, to dispersal, predation, and, finally, seedling establishment. Therefore, the whole process is not easy to approach given the great number of variables involved and the difficulties to establish relationships among them. Consequently, most studies focus on specific stages, or in some of the variables involved (see the review by [1]), while few have tried to provide a "complete picture" of the whole story (but see [2–4]).

In addition to the inherent complexity that results from the interdependence of several sequential processes, the interaction among the behavior of seed dispersal agents (e.g. depredating or caching seeds) and environmental factors (e.g. facilitation effects) may also strongly influence the success of the recruitment process [1, 2, 5–7]. We know, for example, that the behavior of animals such as scatter-hoarding rodents may strongly shape the patterns of natural recruitment in many plant species, acting either as seed dispersers or predators [8,9]. From an animal-centered perspective, seed attributes, such as the species or size are known to influence the dispersal vs. predation decision [10–13]. Likewise, environmental variables such as shrub cover [10, 14, 15], or intra-guild competition (e.g. ungulate presence in [16–18]) are known to influence rodent’s foraging decisions. From the plant perspective, it has been documented that recruitment likelihood may depend on seed abundance (see [2]), microhabitat availability (e.g. facilitation; [6, 19, 20]) and seed/seedling consumption by granivores and herbivores [2, 16,21]. However, most studies focused on seed dispersal behavior have rarely tested its real contribution to seedling recruitment (but see [22–24]) while those studies centered in plant recruitment have rarely provided evidences on the contribution of foraging behavior by seed predators/dispersers in this process.

To provide an integrative view, it is necessary a sequential-stage approach where the different stages, from seed production to seedling recruitment, are considered in light of the interplay among both environmental and behavioral traits [22, 25]. A spatially explicit design can be helpful to improve our knowledge on the relationships between these variables, and their contribution to the patterns of seedling recruitment. Measuring a collection of environmental (e.g. seed availability, shrub cover or presence of ungulates) and behavioral (e.g. seed dispersal, seed predation, seed caching) variables in fixed points at a small spatial scale may allow to assess their spatial variation and relationships over short distances. It is known that environmental variables may vary dramatically in small spatial scales [26], and some studies have recently questioned if they have a fixed effect over disperser’s behavior and seed fate, or their effects are strongly context dependent varying in space and time [13, 24, 25, 27]. This suggests that animal foraging behavior may be also highly heterogeneous at a small spatial-scale and may partly account for the heterogeneous spatial patterns in seedling establishment in combination to environmental features [6, 28, 29]. Moreover the relevance of environmental vs. behavioral variables for seedling recruitment may be expected to be highly context dependent and vary in different ecological scenarios or according to the tree species involved. In fragmented landscapes with low vegetation cover the presence of shrubs may play a key role in facilitating seedlings establishment (e.g. providing shelter to seed dispersers in [17, 30], ameliorating environmental conditions in [6, 19, 31, 32]) while in more continuous forests, seedlings establishment may be more dependent on seed availability [4], or the presence of gaps in the vegetation cover [33]. Similarly, the recruitment of seedlings of co-occurring tree species may be mediated by species-specific differences in the behavior and preferences of seed predators (see for the effects of differences in seed size [34]) or dispersers (see for effects of differences in phenology [13]), and the relative tolerance of these species to environmental stresses [35].

The recruitment of oak species in Mediterranean forests is an excellent study model to assess the role of the interactions among animal's behavior and environment on the establishment of seedlings. Oaks produce big sized seeds (acorns), which are highly nutritious and are a precious food source for many animal species such as rodents (e.g. the wood mouse, *Apodemus sylvaticus;* [22, 36]) or ungulates (e.g. the wild boar, *Sus scrofa;* [18]). Wild boars may have a strong impact on oak's recruitment, since they consume great amounts of acorns from the ground [18], and also kill many emerged seedlings with their rooting activity [21]. On the other hand rodents do not only consume a lot of acorns, but also disperse them actively along the forest microhabitats [16, 22, 36],and eventually cache them as food reserves, potentially aiding their germination [37]. However many of the seeds placed in buried caches end up being pilfered by other rodents, which may consume them immediately or cache them in a new location (for a review, see [38]). The main objective of this study was to assess the variability of environmental and behavioral factors involved in the interactions between seed-dispersing rodents and oaks in a mature Mediterranean mixed oak (*Quercus ilex*, *Q*. *pubescens*) forest, in order to analyze their relevance and how they are inter-connected and linked to the patterns of seedling establishment. Specifically, we assessed the variability of the environmental variables involved in the recruitment process (acorn abundance, tree canopy and shrub cover, and rodent abundance) and the behavioral ones (seed predation/seed dispersal patterns and cache pilferage activity by rodents, and wild boar activity). Then, we tested the influence of environmental features on rodent behavior and we determined the contribution of these two sets of variables in the patterns of seedling recruitment

## Methodology

### Study site and species

The study was conducted in Can Balasc, at the Collserola Natural Park (Barcelona, NE Spain; 41°24’N, 2°6’E), a typical Mediterranean-type coastal massif, where the dominant oak species are the evergreen Holm oak (*Quercus ilex*), and the winter-deciduous Downy oak (*Quercus pubescens*), two oak species that mature acorns in one year [39]. At a habitat scale the forest understory is dominated by a fairly continuous shrub layer (*Viburnum tinus*, *Lonicera implexa*, *Clematis flammula*, *Arbutus unedo*, *Bupleurum fruticosum*, *Rhamnus alaternus*, and *Phillyrea latifolia*); ground-layer vegetation dominated by *Hedera helix* (see [40]). Acorns are the food source for many mammals such as the wild boar (*Sus scrofa;* [18]), very abundant in the park [40], and scatter-hoarding rodents (e.g. *Apodemus sylvaticus*, *Mus spretus)* which predate and disperse them actively (see for the study area [13]). Oak seedlings emerge at March-April (see [41]).

### Experimental design

To perform a spatially-explicit approach we delimited the study area by marking 132 fixed points with tagged wooden stakes forming a grid with a 10-meter separation among them, and georeferenced with a sub-metric GPS (Geo Explorer 6000 Series XH, Trimble). The location of the grid was selected to ensure that a half of the points (65) were placed inside an area where wild boars were excluded by means of a 2-meter high metallic fence, while the rest of the points (67) were placed outside the exclosure. From November 2010 to June 2011, we monitored different environmental and behavioral variables involved in the process of natural regeneration at each of the marked points. The variables measured were:

#### i) Tree and shrub cover

Although the shrub cover may seem generally continuous at a habitat scale, it is heterogeneous at the fine spatial scale considered in the study, with some sampling pointsin much more open sites than others. Hence, at each marked point we determined visually the percentages of shrub cover and tree canopy cover on a circular area with 1-meter radius from the wooden stake.

#### ii) Availability of acorns on the ground

We monitored the availability of acorns on the ground every 25 days (9 samplings) from November to June. At each sampling, we counted all the acorns on a circular area of 1-meter radius from every stake, distinguishing species membership (*Q*. *ilex*, *Q*. *pubescens*) and condition (sound or infested by insects).

#### iii) Abundance of rodents

We monitored the rodent abundance in the study area every 25 days (9 different campaigns) from November to June, using Sherman live-capture traps (23.5 x 8 x 9 cm; HB Sherman Traps Inc. Tallahassee, Florida USA). In each campaign, we placed 132 traps (one per stake) baited with a mixture of flour and tuna in oil, a piece of apple to avoid dehydration, and a handful of hydrophobic cotton so that the captured rodents could make a nest to keep warm until they were marked and released (see for this methodology [17]). The traps were set for four consecutive nights at sunset (i.e. 20:00 hours GMT) and were checked every morning at sunrise (i.e. 07:00 hours GMT) to minimize the captivity duration. Captured rodents were marked with asubcutaneous microchip (MUSICC, AVID Identification Systems Inc) using a specialized syringe (AVID SUDS monoject) and immediately released at the exact same point where they were caught.

#### iv) Wild boar rooting activity

We estimated wild boars’ rooting activity by noting the percentage of the ground surface affected by rootingevery 25 days (9 different surveys) along the entire seeding season within a circle of a 1-meter radius from each stake located outside the enclosure to exclude ungulates.

#### v) Predation/dispersal patterns of acorns by rodents

The patterns of seed predation and dispersal by rodents were measured with marked acorns in 40 feeding plots distributed across the study area (20 points out of the exclosure and 20 points inside). Each feeding plot consisted of a wire cage with a mesh size of 5x5 cm in which the marked acorns were placed, ensuring that only scatter-hoarding rodents would have access to them [13]. Acorns were marked with a 40-cm-long wire with a flagging tape [42]. We placed 320 tagged acorns along the 40 sites so that each feeding plot had eight acorns: four of *Q*. *pubescens*, and four of *Q*. *ilex*. We visited the feeding plots at 25 days intervals from the beginning to the end of the seeding season (7 samplings). At each visit we recorded the acorns predated *in situ* and tracked those dispersed outside the cage, noting the dispersal distance, microhabitat at destination, and their fate (predated or intact). All marked acorns were replaced in the feeding plots by new fresh ones at each monitoring, maintaining the same proportion of species. Thus, by the end of the study we had monitored the fate of 2240 acorns (= 320 acorns x 7 revisions). Those acorns that were dispersed but not eaten were left on the field and checked during the following samplings, until they were predated or re-dispersed to a new location [13].

#### vi) Pilferage of acorn caches

The patterns of cache pilferage by rodents were monitored by caching 268 acorns on 67 marked points (34 inside the exclosure and 33 outside). On each point, we artificially cached 4 ripe acorns (2 of *Q*. *ilex* and 2 of *Q*. *pubescens*) at 3 cm deep simulating the caches made by rodents [22], at a distance of 0.5 meters from the stake following the cardinal points to enable their monitoring. Cached acorns were monitored every 25 days along the entire seeding season (9 visits) and we registered whether they were intact, pilfered, desiccated, germinated, or had produced a seedling.

#### vii) Seedling emergence

Finally, once emergence period had finished (May) we measured the emergence of oak seedlings at every marked point. We counted all the emerged oak seedlings of each species (*Q*. *ilex* and *Q*. *pubescens*) in a surface of 1-meter radius from the stake of all sampling points. In addition, we tagged all the seedlings with plastic ties and returned next year to check seedling survival. It should be stressed that this study was performed over a single year, so that the results do not account for interannual variability in climatic conditions (such as temperature and rainfall). However, previous studies have emphasized that in these oak forests early recruitment processes are of paramount importance to explain the regeneration patterns observed (see [4])

### Data analysis

To validate the consistency of the monitored variables and to consider their categorization, we initially performed a descriptive analysis of each variable noting the minimum and maximum values, the mean and standard deviation, the number of observations, the missing values, and their distribution. We also calculated the coefficient of variation (CV) for each factor as a measure of their spatial variability among the sampling points along the study area.

We used generalized linear models (GLM) with a binomial distribution and a logit link function to analyze the factors affecting rodent’s foraging behavior: number of acorns manipulated, number of acorns dispersed, and number of acorns predated (over the 56 tagged acorns supplied in each feeding plot along the study), and the number of caches pilfered (over the four artificial caches placed in each sampling point). Moreover, we used GLM with a normal distribution to assess the factors affecting the acorn dispersal rates (number of acorns dispersed relative to the number of acorns manipulated, including dispersed and consumed *in situ*, for each point), and species preferences (number of manipulated *Q*. *ilex* acorns relative to the total number of manipulated acorns, per point). Finally, we also used GLM with a negative binomial distribution and a logit link to assess the factors affecting the emergence of oak natural seedlings (total, and for each *Quercus* species separately) at the end of the season. To assess the existence of over-dispersion in the models we used Pearson’s chi-square information criteria test. In all the different GLM we used the vegetation structure (shrub cover and tree canopy cover), abundance of acorns on the ground, abundance of rodents, and wild boar rooting activity, as fixed independent variables. For the models assessing the emergence of oak seedlings (total and for each *Quercus* species) we only considered the abundance of sound acorns since the infested ones have extremely low probabilities of germinating and producing viable seedlings [43]. In addition to the abovementioned independent variables, in this particular model we also included the pilferage rates of caches as an explicative variable to directly test for its potential effect on seedling establishment. Moreover, for the models assessing seedling emergence of each species separately we also introduced a variable representing the relative abundance of sound acorns of each species (*Q*. *ilex* acorns / *Q*. *pubescens* acorns) on the ground.

The selection of significant variables for all the models was carried out by backward automatic procedure, assuming a signification level of p < 0.05. To build the models we used categorized values for canopy cover (< 85% and > = 85%), shrub cover (< = 25%, 25–85% and > = 85%), availability of acorns (< = 1 acorns, 1–4 acorns and > 4 acorns), and wild boar rooting activity (0%, 0–10% and > 10%), since it allowed an easier interpretation of the results. To categorize these variables we aimed for an equitable distribution of the observations among the different categories of each variable, also considering a biological criterion to establish each category. Rodent's abundance (0 to 13) and number of caches pilfered (0 to 4) remained as numerical variables as they were key factors for the models, and we believed their categorization could reduce the accuracy when testing their effects. The use of categorized variables did not affect the results since very similar models were selected when continuous values were used.

To evaluate any spatial dependence we used a test of covariance based on the ratio of residual likelihoods to compare each GLM (complete independence of Spatial Effect) with its counterpart introducing the sampling point as a residual random factor. Since no differences were found between the models in any case we finally used the simpler model (i. e. the GLM without the random factor). The significance of the fixed variables was assessed by type III test of fixed effects, and their effect was reported from the Parameter Estimates for the continuous variables (number of rodents and pilfered acorns), and from the Estimated Odds ratio and the Estimated Least Square Means (including a test of the differences of all categories, taking into account the Tukey adjustment for multiple comparison). Data analysis was performed with SAS software version 9.3 [44].

### Ethical standards

This study was conducted in the protected forest of Can Balasc, in the Collserola Natural Park, in accordance to the current Spanish legislation and with a permit of the Collserola Natural Park authorities (Num: 808). Such permit was obtained after the revision and approval of the experimental design and capture methodology by the Natural Park biologists. We did not approach other ethics committees, since the park authorities are the ones in charge of preserving the fauna and flora of the park, and assuring the animals’ welfare. Hence, the approval from an external animal ethics committee was not needed.

## Results

The study area presented a dense and quite continuous tree and shrub canopy cover (Mean ± SE respectively, 73 ± 3% and 61 ± 3%), with a low coefficient of spatial variation (respectively, CV = 0.41 and CV = 0.49), although both ranged from 0 to 100%. Conversely, the mean abundance of acorns on the ground (3.4 ± 0.5 acorns m^-2^) was much more variable in space, ranging from 0 in some sites to 44.9 acorns m^-2^, and thus presenting a high CV (1.68).

We captured 264 wood mice, *Apodemus sylvaticus*, and 2 Algerian mice, *Mus spretus*, over the nine trapping campaigns. The average number of mice captured across the study area was 3.4 ± 0.2 per trap, ranging from 0 up to 13 individuals. Although there were some points with no captures, rodents were evenly distributed at the entire study area so that spatial variability of rodent abundance was quite low (CV = 0.76).

At the end of acorn monitoring, rodents had manipulated (predated *in situ* or dispersed) 169 of the 2240 tagged acorns offered (4.2 ± 0.6 acorns per cage). Acorn manipulation showed a moderate variability across the study area (CV = 0.85), and was positively influenced by shrub cover (F_32_ = 9.7, p < 0.001), and the interaction between rodents’ abundance and acorn availability (F_32_ = 7.0, p = 0.003): i.e. rodent abundance increased acorn manipulation, except in those points with very few acorns naturally available. A similar pattern was also observed for the models considering just the acorns dispersed or predated (results not shown).

At the end of the experiment, 86.4% of the acorns manipulated by rodents had been dispersed from the cages (6.5 ± 0.9%). Seed dispersal rates (number of dispersed seeds respect those manipulated) ranged from 0 to 100% but showed very low variability along the study area (CV = 0.30). Rodent abundance had a positive effect on the dispersal rates, especially in places with lower tree canopy cover (F_18_ = 5.2, p = 0.035) and with higher abundance of acorns (F_18_ = 5.6, p = 0.013). On the other hand, rooting activity by wild boar strongly reduced the dispersal rates of acorns by rodents, especially in those places with denser tree canopies (F_18_ = 4.0, p = 0.036). Moreover, the interaction among rodent’s abundance and wild boar rooting (F_18_ = 6.1, p = 0.009) revealed that rodent’s positive effect on dispersal rates shifted to a negative effect in those places rooted by wild boars.

Interestingly rodent’s dispersal activity changed the spatial patterns of seed shadows concerning shrub cover. Rodents biased seed transfer towards open places, as most acorns dispersed (95.9%) came from site under a shrub and, 44.4% of them (N = 52) were dispersed to open places. By contrast only 4.1% (N = 5) of the seeds dispersed by rodents were removed from open places, and only 2 of them were transferred to a site under shrub cover. Additionally, rodents did not show any significant differences in their preference for each species, showing similar patterns across the study area (CV = 0.56). However, the GLM revealed a positive effect of acorn abundance on the preferential selection of *Q*. *ilex* over *Q*. *pubescens* acorns mediated by shrub cover (F_17_ = 3.8, p = 0.021). Most dispersed acorns (93.9%) were finally consumed, and only 6.1% of dispersed acorns escaped predation.

Pilferage activity was widespread and generally high, as 46.3 ± 3.6% of the artificial caches placed were pilfered by rodents, (CV = 0.77). Pilferage rates were directly related to rodent abundance (F_65_ = 5.1, p = 0.027; β = 0.14 ± 0.06), but not influenced by shrubs, tree canopy cover or rooting by wild boars

**Fig 1:**
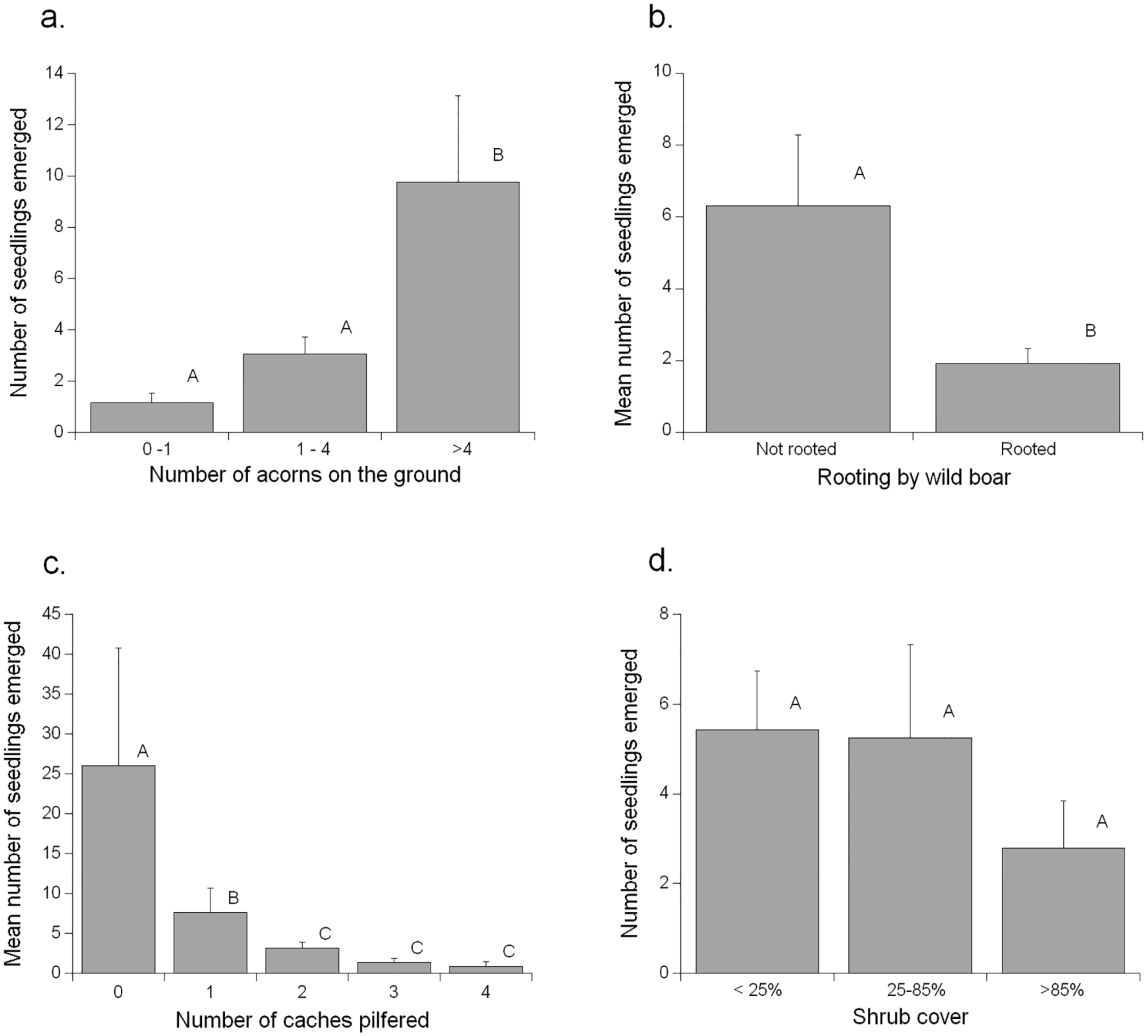
Effects on the early recruitment of oak seedlings. Effects on oak’s recruitment of the number of acorns in the ground (a), wild boar rooting activity (b), cache pilferage by rodents (c), and shrub cover (d). Different letters represent statistical differences according to the Fisher LSD test. Results represented are mean values ± standard errors.

Seedling emergence (1.5 ± 0.4 seedlings m^-2^) presented the highest spatial variability across the study area (CV = 3.1) among all measured variables, ranging from 0 to 45 emerged seedlings m^-2^. At the end of the season the total number of emerged seedlings of each species was similar (273 of *Q*. *ilex* and 268 *Q*. *pubescens*). Overall seedling emergence was positively affected by the number of acorns available on the ground (F_57_ = 10.8, p < 0.001)(Fig 1a), and negatively affected by the rooting activity of wild boar (F_57_ = 3.6, p = 0.035)(Fig 1b). More interestingly, the pilferage activity by rodents had a strong effect on the number of seedlings emerged (F_57_ = 27.6, p < 0.001; β = -0.72 ± 0.14)(Fig 1c). By contrast, other environmental variables like shrub cover (Fig 1d), or other behavioral variables like acorn dispersal or predation, did not influence seedling emergence. These patterns also held true when considering the seedlings of each species separately, also showing strong negative effect of pilferage activity over natural seedling emergence of each species (*Q*. *ilex*: F_51_ = 9.2, p = 0.004, β = -0.49 ± 0.16; *Q*. *pubescens*: F_53_ = 18.6, p < 0.001, β = -0.98 ± 0.23). Nevertheless, seedling survival after 1 year was higher (t_100_ = 2.49, p = 0.014) for *Q*. *ilex* (37.36%) than for *Q*. *pubescens* (24.63%).

## Discussion

The main contribution of this study is to show that seedling recruitment in Mediterranean mixed oak forests may show a very high spatial variability, not just mediated by a facilitationor nursingeffect of shrub or tree location as often suggested (see examples in [45]), but mostly driven, in this more continuous forests, by the spatial variability in the seed shadows and the behavior of seed predators and dispersers (i.e. cache pilferage by scatter-hoarding rodents and the effects of wild boar activity). Moreover, we reported, apparently for the first time, the effect of an interesting animal-to-animal interaction influencing seed survival and recruitment likelihood: i.e. wild boar’s presence modified rodent’s seed dispersal/predation behavior, as they shifted from seed dispersers to the consumption of acorns *in situ* in those places rooted by boars. The importance of animal-to-animal interactions in the behavior of scatter hoarding rodents is increasingly receiving more interest (e.g. [16, 46]) and providing evidences of relevant consequences for seedling recruitment of intra-guild competition [47], [48], or the consequences of seed dispersers’ fear to predators [49].

Seedling emergence in our study area was not related to the distribution of tree or shrub cover (Fig 1d), differing from the results of other studies in Mediterranean-type forests that have stressed a ‘nurse effect’ of shrubs on recruitment by means of different effects: i) the enhanced handling and selection of seeds by seed-dispersing rodents [10, 15, 17], ii) suitable abiotic conditions for seed germination and seedling emergence [6, 19, 31, 32], and iii) protection of seedlings from ungulates [21, 50]. However, it should be stressed that these results were often derived from studies conducted in areas were vegetation cover is scarce and often distributed in a patchy pattern. In that scenario, trees and shrubs may certainly play a major role in providing shelter to seed dispersing rodents [15, 17], or facilitating seedling emergence and survival by reducing environmental constraints (e.g. radiation and water stress in [6, 31, 32, 45]). Conversely, the more dense and homogenous tree canopy cover in our study area may provide a general shelter to seedlings and thus make recruitment patterns to be more influenced by seed abundance and less dependent on the availability of shrubs as suitable microsites (see for similar results [2, 4, 51, 52]). Indeed, our results showed that seedling emergence was strongly and positively related to seed abundance on the ground (Fig 1a), while scatter-hoarding rodents changed the seed shadows from shrubby towards open sites. This suggests that preferred site for seed-dispersal by rodents (i.e. under shrubs or to forest gaps) is not a fixed behavior but may be highly context dependent. Other studies have also documented a directed seed-dispersal by rodents to open sites [22, 53], that may be explained by the lower pilferage rates in these uncovered sites due to the higher predation risk in absence of shelter. Moreover, the behavior of rodents observed in our study area may be extremely interesting for oak recruitment, considering that previous studies in these forests have shown that seedling establishment and growth may be ultimately constrained by the excessive shadow and competence provided by the tree layer. Thus, seedling recruitment could benefit from the germination of (rodent-dispered) acorns in gaps receiving mid radiation levels [33, 54, 55].

The lack of a key effect of environmental constraints in the early seedling recruitment stages in our study area, as suggested by the absence of nursing effects, probably turned seed availability, mediated by rodent behavior (i.e. pilferage and acorn predation), to be the most relevant drivers in the process of seedling establishment (see also [4] for the effects of acorn crop size). Some studies have documented that pilferage may increase with vegetation cover, as rodents are more abundant and active in these sites [22, 56], a pattern supported by the strong correlation between pilferage rates and rodent abundance found in our study area. However, one important finding of our study is that the activity of rodents as pilferers of cached seeds may ultimately influence the recruitment patterns of oaks (Fig 1c). Rodents’ pilferage activity has been traditionally approached from a behavioral perspective [38, 56–58]. So far, pilferage has been understood as the “theft of cached food to another animal”, but, to our knowledge, its impact on plant’s reproduction has been less often considered (but see [23, 32]). Our study provides direct evidences that pilferage may certainly have this broader dimension, as we found a negative relation between pilferage activity and recruitment: i.e. those places where rodents stole more caches had lower number of emerged seedlings. We cannot ensure whether pilfered caches were finally consumed by the rodent, or re-cached in another location, which would have no effects on the reproductive success of the plant but in its spatial distribution. Anyway, these results reinforce the hypothesis that in our forest seed availability is the most limiting factor for recruitment. In fact, seed abundance may also play an important role for seedling emergence in relation to rodent abundance, since great seed crops have been found to satiate rodents, increasing the recruitment chances for the plant [59]. Nevertheless, in our study area we did not find a direct effect of rodent abundance on seedling emergence, probably due to the small spatial scale considered (i.e. the home range of a wood mouse individual may include several sampling points where rodent abundance and seedling emergence were measured).

In addition to seed pilferage, seedling establishment suffered a direct and indirect negative impact from the foraging activity of wild boars (Fig 1b), an abundant species in our study area [40]. Wild boarsareknown to directly reduce oak’s recruitment chances by consuming great amounts of acorns [18], and killing the emerged seedlings when rooting [21]. Surprisingly, we also found an indirect negative effect of the presence of wild boars as it switched the foraging behavior of rodents, decreasing dispersal and increasing the *in situ* consumption of acorns (see also [16]). Certainly, in terms of optimal foraging theory, this change in behavior is reasonable in order to reduce the probability of intra-guild competition by wild boars (e.g. acorn predation and destruction of caches). Rodent’s response to competition by modifying their behavior may have important consequences for the fate of the seeds they use (i.e. consume vs. disperse), as described for fear-induced behavioral responses (e.g. [49]). These results provide new evidence of the importance of animal-to-animal interactions on the behavior of rodents, and the consequences of these interactions for the patterns of seed dispersal vs. seed consumption. Moreover, this shift in the outcome of the rodent-seed interaction represents a good example of how ecological interactions in nature may be non-monotonous, which has been found to contribute in maintaining stable and complex ecological networks [60].

Concerning the two oak species, the effects of environmental and behavioral variables on the recruitment of *Q*. *ilex* and *Q*. *pubescens* seedlings were very similar, except for a slightly higher preference of rodents for the acorns of *Q*. *ilex*, mediated by the largest acorn crops of this species, and a higher survival of the established seedlings of this oak. These results are in line with previous studies that have shown a key role of the largest acorn crop sizes produced by *Q*. *ilex* both for a preferential manipulation of their seeds by rodents [49], and for the more abundant seedling recruitment of that species in comparison to *Q*. *pubescens* [4, 39]. The benefits of a largest seed availability of *Q*. *ilex* for seedling recruitment is complemented by the higher survival rates of this species, as shown by our results, in the current environmental conditions of this mixed oak forest types [61], while *Q*. *pubescens* seedlings would be favored under shadier conditions of more mature forest structures [35].

In Mediterranean-type communities, the spatial pattern of seedling recruitment of late- successional species, such as oaks, has been traditionally assumed to depend on the facilitationor nursing effect of the tree and shrub layers (see among others: [33, 62–64]). Yet, we have shown that in more mature forests, this protective effect may be overridden by the interplay among seed availability, rodents' behavior and wild boar rooting. In sum, our results suggest that the relevance of facilitation for seedling recruitment and the role that rodent’s activity may play as seed dipersers/predators can be highly context dependent on forest structure, varying from a great importance of facilitation in low density savannah-like landscapes to a minor role in dense forests where rodents may even contribute to the dispersal of seeds to forests gaps. Considering how the interactions among behavioral and environmental variables that we report here may affect seedling establishment, we suggest that further studies dealing with forest dynamics should incorporate this broader view to better grasp the complexity of the processes involved in forest regeneration, avoiding studies focused separately in environmental or behavioral traits. Furthermore, it would be very interesting to assess whether the interactions described here are consistent in time and may ultimately contribute to determine the forest structure.
